# Postoperative Management with a Polyurethane Cup Containing an Oxygenated Oleic Matrix in Nipple-Sparing Mastectomy with Immediate Reconstruction: A Single-Center Retrospective Observational Study

**DOI:** 10.3390/jcm15083092

**Published:** 2026-04-17

**Authors:** Giulia Deguidi, Lorenzo Bertoldi, Marina Caldana, Sara Mirandola, Valeria Tombolan, Giuseppe Biondo, Alessia Scirpoli, Francesca Pellini

**Affiliations:** UOC Breast Surgery, Department of Surgery and Dentistry, Azienda Ospedaliera Universitaria (AOUI) Verona, P.le Stefani, 37126 Verona, Italy; lorenzo.bertoldi@aovr.veneto.it (L.B.); marina.caldana@aovr.veneto.it (M.C.); sara.mirandola@aovr.veneto.it (S.M.); valeria.tombolan@aovr.veneto.it (V.T.); bndgpp@gmail.com (G.B.); alessiascirpoli@gmail.com (A.S.); francesca.pellini@aovr.veneto.it (F.P.)

**Keywords:** nipple-sparing mastectomy, immediate breast reconstruction, reactive oxygen species (ROS), skin flap vitality

## Abstract

**Background/Objectives**: Nipple-sparing mastectomy with immediate reconstruction is a preferred option for selected patients undergoing prophylactic or therapeutic mastectomy. Optimizing postoperative wound care is essential to support healing, preserve the nipple–areola complex, and prevent delays in oncologic treatments. This retrospective observational study aimed to evaluate the clinical outcomes associated with the use of the NovoX^®^ Cup medical device in post-NSM surgical wound management, assessing clinical–surgical outcomes and quality of life (QoL). **Methods**: We conducted a retrospective observational study on 54 patients who underwent NSM with immediate reconstruction at AOUI Verona between January 2025 and January 2026; Novox^®^ Cup was applied intraoperatively and changed every 48 h according to protocol. Surgeon-reported outcomes were assessed by the skin flap viability scale and the complications by Clavien–Dindo classification. Patient-reported outcomes were assessed via the Wound-QoL17 questionnaire at 7, 30, and 90 days. Clinical outcomes were supported by photographic documentation. **Results**: Mean age was 51.5 years; BMI averaged 23.9 kg/m^2^. Local complications occurred in 30.4% of cases (infections 12%, dehiscence 10%, seromas 4%). Mean healing time was 15 days, with 87.4% of patients having drains removed by day 14. One patient required surgical revision, and one (1.8%) experienced delayed adjuvant therapy. Wound-QoL17 responses showed minimal discomfort and high satisfaction. Clinical evaluation revealed favorable wound appearance and preserved NAC perfusion within 48 h. **Conclusions**: Novox^®^ Cup appears effective in supporting wound healing and NAC preservation after NSM, with high patient satisfaction and minimal treatment delays. Its integration into postoperative care may enhance outcomes and maintain oncologic timelines.

## 1. Introduction

Breast cancer remains the most frequently diagnosed malignancy and the leading cause of cancer-related mortality among women worldwide. According to recent global estimates, over 2.3 million new cases are diagnosed each year, representing approximately 11.7% of all cancers [1]. Advances in screening programs, early detection, and multidisciplinary treatment approaches have significantly improved survival rates, shifting the focus toward optimizing oncologic safety while preserving quality of life and aesthetic outcomes.

Among surgical treatment options, nipple-sparing mastectomy (NSM) has gained increasing acceptance over the past two decades. This technique allows for the complete removal of breast glandular tissue while preserving the skin envelope and nipple–areola complex (NAC) [2], thereby offering superior cosmetic results compared with traditional mastectomy approaches. NSM is now considered a safe and effective procedure in selected patients with early-stage breast cancer or high-risk genetic mutations, provided appropriate oncologic criteria are met.

Breast reconstruction following mastectomy has also evolved considerably, particularly with the development of prepectoral and subpectoral implant-based techniques. Subpectoral reconstruction, the traditional approach, involves placing the implant beneath the pectoralis major muscle, which can provide enhanced soft tissue coverage but is often associated with increased postoperative pain, animation deformities, and longer recovery times. In contrast, prepectoral reconstruction—where the implant is placed above the muscle, directly beneath the skin flap and supported or not [3] by acellular dermal matrices (ADM) or synthetic meshes —has emerged as a viable alternative. This technique offers several advantages, including reduced postoperative discomfort, preservation of muscle function, and more natural breast contour, especially when performed in carefully selected patients with good-quality skin flaps [4]. Despite technical refinements, NSM remains vulnerable to ischemic complications due to the thinness of mastectomy flaps and the disruption of subdermal vascular networks. Borderline perfusion states (clinically classified as mild or moderate suffering) represent a critical window during which targeted postoperative management may influence the final outcome. Therefore, interventions aimed at supporting microcirculation in the early postoperative phase are of particular clinical interest.

The expanding indications and improved reconstructive techniques associated with NSM and implant-based reconstruction have underscored the importance of managing postoperative healing, minimizing complications, and supporting optimal tissue recovery—critical aspects for achieving both oncological and aesthetic goals [5]. In this context, novel adjunctive therapies aimed at enhancing wound healing, reducing local inflammation, and preventing infection are being increasingly explored in the postoperative setting. At the UOC Breast Surgery of AOUI Verona, approximately 73% of patients who are candidates for mastectomy undergo immediate breast reconstruction. This high rate reflects the multidisciplinary approach and the center’s commitment to combining oncologic safety with optimal aesthetic and functional outcomes, especially in nipple-sparing mastectomy cases.

A growing body of experimental evidence suggests that reactive oxygen species (ROS) are not merely cytotoxic metabolic by-products but also play crucial roles in cell signaling and regulation. This apparent paradox may be explained, at least in part, by the concentration-dependent effects of ROS, where low levels contribute to physiological processes while high concentrations are associated with cellular damage. Biochemical and metabolic studies of the microenvironment in chronic skin lesions have demonstrated the essential role of oxygen in tissue repair processes, including cell proliferation, angiogenesis, collagen synthesis, and defense against pathogenic microorganisms [6]. Neutrophils and macrophages recruited by inflammatory mediators produce ROS at the wound site, which act as secondary messengers for both tissue-resident cells (such as fibroblasts) and immune cells involved in the healing process [7].

Interestingly, similar beneficial effects have been observed following the exogenous application of ROS to wounds [8]. These applications did not produce the expected oxidative damage, likely due to the upregulation of cytoprotective genes and the activation of endogenous antioxidant defense mechanisms [7]. The clinical efficacy of oxygen therapies in chronic wound management is well established, with treatments such as hyperbaric oxygen therapy and oxygen–ozone therapy routinely used in practice. The mechanism of action of oxygen-enriched oil formulations includes not only the controlled release of ROS but also a pH-lowering effect caused by the micro release of carboxylic acids upon interaction with the aqueous component of wound exudate. Maintaining a slightly acidic wound environment is beneficial in preventing chronicity and creating unfavorable conditions for microbial proliferation.

Based on these biological premises, **Novox^®^ Cup**, a topical medical device consisting of a polyurethane dome internally coated with an oxygen-enriched oleic matrix capable of releasing reactive oxygen species (ROS), has been developed. This innovative dressing is designed to maintain intimate contact with the surgical wound, creating a moist and slightly acidic microenvironment that promotes tissue regeneration, angiogenesis, and antimicrobial defense. Its anatomical shape allows for even distribution of ROS over the wound surface while providing a mechanical barrier against external contaminants.

## 2. Materials and Methods

Considering the growing use of nipple-sparing mastectomy combined with immediate implant-based reconstruction—either in prepectoral or subpectoral planes—and the importance of optimizing postoperative healing, we designed a single-center observational study to evaluate the clinical utility of Novox^®^ Cup.

This retrospective observational study aimed to evaluate the clinical outcomes associated with the use of the ***NovoX^®^ Cup*** medical device in post-NSM surgical wound management, assessing clinical–surgical outcomes and quality of life (QoL).

1. **Primary Objective**

The primary objective was to evaluate the efficacy of the ***NovoX^®^ Cup*** advanced dressing in promoting proper healing of the skin flap and nipple–areola complex (NAC) in patients undergoing **nipple-sparing mastectomy (NSM)** with immediate reconstruction, by measuring the incidence of postoperative skin complications and the degree of flap viability according to the adopted standardized scale.

2. **Secondary Objectives**

- **Evaluate the impact of the treatment on the quality of life (QoL)** of patients by administering the **Wound-QoL17** questionnaire at the same follow-up intervals.
- **Monitor the incidence, type and severity of postoperative complications** according to the **Clavien–Dindo** classification, within 90 days and up to 365 days after surgery.
- Document the evolution of the surgical wound, skin flaps and scar throughout the follow-up through standardized digital imaging.
- Explore the association between clinical and anamnestic variables (age, BMI, smoking habit, comorbidities, previous radiotherapy or breast surgery, duration of surgery) and skin healing outcomes.

The study was conducted at the UOC of Breast Surgery AOUI Verona and included a total of 54 patients treated between January 2025 and January 2026. Of these, 36 patients underwent bilateral prophylactic nipple-sparing mastectomy, while 18 patients underwent unilateral nipple-sparing mastectomy for histologically confirmed breast cancer.

A total of 90 ***Novox^®^ Cup*** devices were applied in the immediate postoperative period following implant-based breast reconstruction, either in a prepectoral or subpectoral plane, depending on surgical indications and intraoperative assessment.

### Study Population

Eligible participants were identified through a review of electronic medical records –*EUSOMA DATABREAST*– and operative reports.

*Inclusion criteria were:*

- Female patients aged ≥18 years
- Underwent nipple-sparing mastectomy for therapeutic or prophylactic indications
- Received immediate breast reconstruction (prepectoral or subpectoral, with or without ADM)
- Application of the ***Novox^®^ Cup*** device during the postoperative period

Regarding the application of the ***Novox^®^ Cup***, the medication was placed directly in the operating room at the end of the surgery. It was not fixed to the skin with any adhesive but was instead kept in place by a supportive post-surgical bra. The device remained in place for 48 h post-surgery, after which the patient was instructed to replace it at home with a new device. The dressing was then removed after an additional 48 h application time. This simple yet effective method contributed to a smooth postoperative recovery process, with minimal discomfort reported by the patients.

For each patient, information relevant to the study was collected. This includes age, BMI, gender, ethnicity, smoking habits, cardiovascular and/or rheumatologic comorbidities, and any usual therapies, within a complete remote medical history, duration of surgery, previous breast surgery, and prior radiation treatment.

All data were collected and securely stored in a dedicated database by the responsible investigators, in full compliance with current data protection regulations and best practices for clinical research conduct.

Digital photographs were obtained at each scheduled follow-up visit for all patients. These images provided qualitative documentation of wound healing progression, supporting the clinical assessments.

Data were collected retrospectively from the patients’ medical records, operative notes, follow-up visits, and nursing documentation.

During the perioperative phase, the literature highlights the pivotal role of the breast care nurse in guiding, informing, and supporting the patient throughout the entire care pathway. Nursing interventions described in the selected articles focus primarily on the preoperative consultation, where effective communication becomes essential to ensure that patients clearly understand each stage of breast cancer treatment. The breast care nurse acts as a key point of reference, offering personalized information, addressing concerns, and fostering a trusting relationship through an empathetic and patient-centered approach.

In this context, the nurse provides not only technical and practical guidance—such as advice on preoperative preparation, the choice of supportive bras, options regarding breast implants, and access to support groups—but also continuous emotional and psychological support. Particular emphasis is placed on the nurse’s role in clarifying doubts related to surgical safety, medication management, and perioperative procedures, thereby reducing anxiety and promoting informed decision-making.

Furthermore, the development and use of educational tools, such as written guides or structured informational strategies, are strongly recommended to enhance patient understanding of the perioperative pathway. Through clear, consistent communication and ongoing assistance before and after surgery, the breast care nurse ensures continuity of care, empowers patients, and supports their preparation for both the surgical experience and postoperative recovery.

The post-discharge follow-up mode was organized as follows:

- The patient was scheduled an outpatient visit 5–7 days after surgery, during which the outcomes from a clinical–surgical point of view were monitored to record data on the viability of the NAC/flap, on complications using the Clavien–Dindo scale and the “Wound-QoL17” questionnaire was administered.
- The patient was scheduled an outpatient visit 30 days after surgery, during which the outcomes from a clinical–surgical point of view were monitored to record data on the viability of the NAC/flap, on complications using Clavien–Dindo scale and the “Wound-QoL17” questionnaire was administered.
- The patient was scheduled an outpatient visit 90 days after surgery, during which the outcomes from a clinical–surgical point of view were monitored to record data on the viability of the NAC/flap, on complications using Clavien–Dindo scale and the “Wound-QoL17” questionnaire was administered.

*The surgeon’s point of view*

The follow-up modality from the surgeon’s point of view will be organized as follows:

- The patient will have the advanced dressing in question placed in the operating room at the end of the surgery (day 0 post operation); this dressing will then be replaced by the medical-nursing staff in the ward after 48 h or on day II post operation.
- Once discharged home, the patient will then replace her after a further 48 h, then on day IV post operation.
- The patient will then be scheduled an outpatient visit 5–7 days after surgery.

The patient will be evaluated in the ward according to the flap viability rating scale (Table 1) illustrated below in day 0-1-2 after surgery and at the first outpatient dressing or between day V and VII after surgery, and any other complications will be recorded according to Clavien–Dindo (Table 2).

The skin flap vitality scale (Table 1) is an internally developed clinical assessment tool, created following a multidisciplinary consensus meeting between breast and plastic surgeons within our Breast Unit. The scale is based on commonly accepted parameters for postoperative skin perfusion assessment (color, temperature, capillary refill, edema, and tissue integrity). Although not externally validated, it was systematically applied across all patients to standardize postoperative evaluation.

*The patient’s point of view*

Postoperative quality of life (QoL) was assessed using the validated **Wound-QoL17 questionnaire (**Table 3**)**, a 17-item patient-reported outcome measure specifically designed to assess the physical, psychological, and functional burden of wounds over the preceding 7 days. Each item was scored using a 5-point Likert scale:

- **0 = By no means**
- **1 = Little**
- **2 = Enough**
- **3 = A lot**
- **4 = Very much**

Higher scores reflect greater perceived burden.

The questionnaire covers multiple dimensions of patient well-being, including:

- **Physical symptoms** (e.g., pain, smell, secretion, sleep disruption)
- **Emotional impact** (e.g., sadness, frustration, anxiety about healing)
- **Functional limitations** (e.g., difficulty moving, climbing stairs, or participating in social and recreational activities)
- **Social and financial burden**

Patients were instructed to complete the Wound-QoL17 on postoperative day 14, coinciding with the typical timeline for drain removal and wound stabilization. Responses were collected anonymously and analyzed using descriptive statistics.

Data were collected from completed questionnaires available in the clinical record during early postoperative follow-up. In cases where the questionnaire was only partially completed, available responses were still included in domain-level analysis, provided at least 75% of items were answered.

These data were used to describe the subjective wound-related experience of patients and to explore possible associations with clinical outcomes such as complication rates, drainage duration, and healing time.

**Table 3:** The “Wound-QoL17” questionnaire is divided into 17 questions as follows:

Very Much	A Lot	Enough	Little	By No Means	Question	In the Last Seven Days…
					My wound hurt	**1**
					My wound smelled bad	**2**
					There was a leak of disturbing material from the wound	**3**
					The injury affected my sleep	**4**
					Wound treatment was a burden on me	**5**
					The injury made me miserable	**6**
					I felt frustrated because the wound is taking so long to heal	**7**
					I worried about my injury	**8**
					I was afraid that the wound would get worse or that new ones would appear.	**9**
					I was afraid to hit the wound	**10**
					I had trouble moving because of the injury	**11**
					Climbing the stairs was difficult due to the injury	**12**
					I had problems with daily activities due to the injury	**13**
					The injury limited my recreational activities	**14**
					The injury forced me to limit my activities with others	**15**
					I felt dependent on the help of others because of the injury	**16**
					The injury was a financial burden on me	**17**

The following clinical and patient-reported outcomes were assessed:

- **Local Complications**: The incidence of postoperative complications was recorded, including:
- **Infection** (defined by clinical signs and/or need for antibiotics or surgical revision);
- **Seroma** (requiring aspiration or delaying wound healing);
- **Wound dehiscence** (partial or complete separation of surgical wound edges).
- **Healing Time**: Defined as the time (in days) from surgery to complete wound closure, with no drainage or dressing required.
- **Drainage Duration**: Duration of surgical drains was recorded in days; prolonged drainage was defined as >14 days postoperatively.
- **Reintervention:** The need for surgical reintervention due to complications (e.g., implant removal, revision surgery) was documented.

**Primary Endpoints**

Incidence of skin flap impairment (NAC and mastectomy flaps) assessed according *to the skin flap viability scale* (scores 1–4) in the following timeframes:

- Day 0 after surgery (operating room)
- Day 1 after surgery
- Day 2 after surgery
- Day 5–7 after surgery (first office visit)

Overall incidence of early (within 30 days) surgical wound-related skin complications after NSM: flap distress, infection, seroma, dehiscence, partial/total NAC necrosis, etc.

**Secondary Endpoints**

1. Wound-QoL17 total score, detected in the same ranges, to quantify the impact on quality of life.
2. Incidence and severity of general postoperative complications graded according to Clavien–Dindo within 90 days and up to 365 days.
3. Time to complete wound healing (defined as complete closure and absence of signs of exudate, infection, or tissue distress).
4. Standardized photographic evolution of the wound and NAC, analyzed at follow-up times.
5. Statistical association between clinical/anamnestic variables and outcomes (complications, flap viability scores, Wound-QoL17).

**Success criteria**

Effective treatment is considered if all of the following criteria are met:

- **Flap vitality**
- Score 1 or 2 on the flap vitality scale within 5–7 days after surgery
- Absence of progression to scores 3–4
- **Complications**
- No major wound-related complications (Clavien–Dindo ≥ III)
- Absence of NAC necrosis (partial/total)
- Absence of infection requiring IV antibiotics
- **Quality of life**
- Improvement or stability of Wound-QoL17 score between 30 and 90 days
- **Complete recovery** within the expected time (30–45 days), with no clinically relevant delays.

**Failure criteria**

Treatment failure is considered if **even one** of the following occurs:

- **Significant flap suffering (score 3–4)** (persistent or progressive).
- **NAC necrosis** (partial or total).
- **Major** wound-related complications (Clavien–Dindo III–IV).
- **Severe infection** requiring drainage or surgical revision.
- **Delayed healing > 60 days** with persistence of exudate or dehiscence.
- **Significant worsening of QoL** (clinically relevant increase in Wound-QoL17).

## 3. Results

A total of 54 patients who underwent nipple-sparing mastectomy with immediate breast reconstruction and postoperative application of the ***Novox^®^ Cup*** device were included in this retrospective study. The mean age of the cohort was 51.5 years, and the mean body mass index (BMI) was 23.9 kg/m^2^ (range 18.6–35.7). All patients were female, as per inclusion criteria. Smoking habits were recorded, with 5.4% identified as current smokers. Comorbidities, including cardiovascular and rheumatologic diseases, were present in 40.3% of patients. Usual therapies prior to surgery were also documented.

Of the total, no patient had previously undergone radiotherapy and only one patient had ipsilateral surgery.

The average duration of the intervention was 150 min. The most frequent surgical incisions (95%) were made at the breast sulcus or radial incisions at the upper-external quadrant.

All patients underwent nipple-sparing mastectomy, followed by immediate breast reconstruction, either prepectoral or subpectoral, with or without the use of the acellular dermal matrix (ADM). The ***Novox^®^ Cup*** device was applied postoperatively in all cases. The initial application lasted for 48 h immediately after surgery in the operating room, with no adhesives used to fix the device; instead, it was maintained in place using a supportive post-surgical bra. Patients were instructed to replace the device at home for an additional 48 h, after which the dressing was removed.

- *Results from skin flap vitality scale:*

A total of **54 patients** undergoing breast surgery with skin flap preservation were retrospectively evaluated postoperatively using the **skin flap vitality scale (grades 1–4)**. This standardized clinical scale assesses flap viability based on color, temperature, capillary refill, edema, and the presence of tissue suffering or necrosis.

**One patient (1.85%)** developed **total nipple–areola complex (NAC) necrosis**, corresponding to **grade 4 vitality impairment**. This represented a severe and irreversible vascular compromise, requiring **surgical assessment and dedicated management**.

**Partial necrosis** was observed in **8% of patients**. These cases involved limited areas of tissue suffering without progression to full-thickness necrosis. All were successfully managed conservatively through **advanced wound dressings and close outpatient follow-up**, without the need for surgical revision.

Regarding flap vitality, **18% of patients** were classified as **grade 2 (reduced but acceptable vitality)**. These flaps exhibited mild and homogeneous color alterations, capillary refill times of 2–3 s, and mild to moderate edema, without signs of irreversible ischemia. In all grade 2 cases, a protocol of **enhanced clinical monitoring combined with the application of the Novox Cup dressings** was implemented. **Complete clinical resolution was achieved in 100% of these patients**, with progressive normalization of flap color, temperature, and capillary refill, and no progression to higher-risk categories.

Only **1.84% of patients** were classified as **grade 3**, characterized by evident signs of moderate flap suffering, including delayed or absent capillary refill, reduced temperature, and areas of dubious vitality. These patients underwent **intensified surveillance and early therapeutic intervention**, including optimization of local wound care and serial surgical evaluation, aimed at preventing progression toward necrosis.

Overall, the distribution of complications demonstrates a **low incidence of severe flap-related adverse outcomes**, with the majority of vascular alterations remaining mild and reversible when promptly identified and treated.

- *Results from Clavien–Dindo classification’s complications:*

Postoperative complications were carefully recorded. Local complications occurred in 30.4% of patients, including infections (12%) seromas requiring aspiration or delaying healing (4%) and wound dehiscence (10%). The mean healing time, defined as the interval from surgery to complete wound closure without the need for drainage or dressing, was 15 days. Notably, 87.4% of patients had their drainage removed by day 14, with a drainage output of less than 30 cc. 

**Figure 1:**
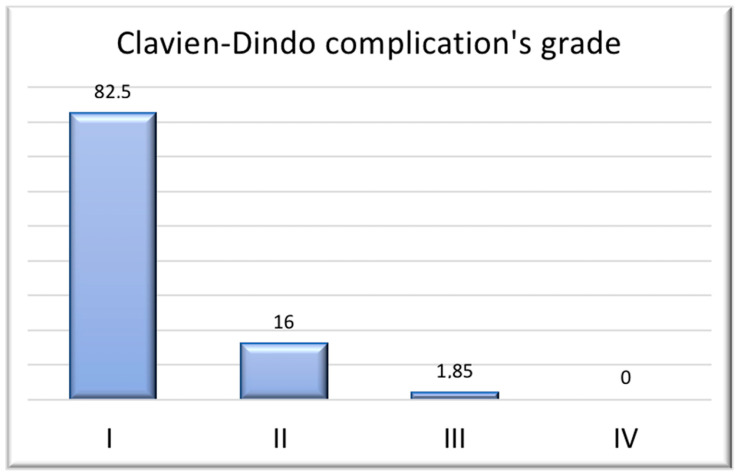
Results from Clavien–Dindo classification’s complications: Most patients experienced either no complications or only minor ones. Specifically, 16% of patients developed seromas that required outpatient needle aspiration, and only one patient had a complication necessitating surgery—necrosis of the NAC, which required its surgical removal.

Surgical reintervention was required in only one patient (Figure 1).

- *Results on Wound-QoL17 questionnaire written by patients:*

**Figure 2:**
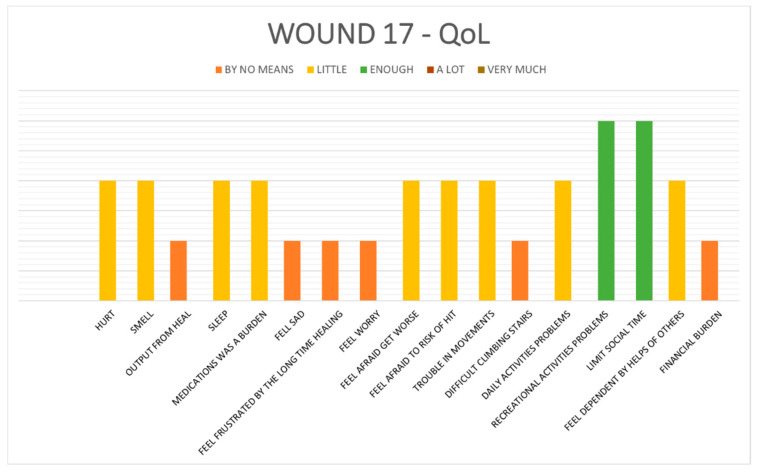
Results from “Wound 17 QoL”: Most patients chose responses in the “By no means” or “Little” categories for nearly all items, suggesting that postoperative wound-related discomfort and limitations were minimal.

Patient-perceived quality of life related to wound burden was assessed through the ***Wound-QoL17 questionnaire*** at postoperative days 7, 30, and 90. All patients completed the questionnaires (Figure 2).

Most patients selected responses within the “By no means” or “Little” categories for nearly all items, indicating that postoperative wound-related discomfort and limitations were minimal. This was particularly notable in areas related to pain, exudate, sleep disruption, emotional distress, mobility, and social interaction. The uniformity of these responses reflects a broadly favorable postoperative experience and suggests that the recovery process was well-tolerated across the patient population.

From a clinical and surgical perspective, early observations corroborated the subjective data. In the immediate postoperative phase, it was possible to appreciate the beneficial effects of the ***Novox^®^ Cup*** on the visual appearance of the mastectomy flaps. Surgeons noted consistent and improved tissue stabilization, with the flaps demonstrating optimal perfusion, minimal edema, and favorable color and texture within the first 48 h after surgery. This early stabilization of the tissue is critical in procedures such as nipple-sparing mastectomy, where vascular integrity and skin viability are essential for both functional and aesthetic success. Furthermore, the use of the ***Novox^®^ Cup*** dressing appears to have contributed to a reduction in localized complications, including ischemic changes and delayed healing of the nipple–areola complex and surrounding flaps. The dressing’s antioxidant properties, combined with its pressure-distributing design, created a favorable environment for tissue regeneration and minimized mechanical stress on the surgical site. This facilitated not only a faster healing trajectory but also more predictable and uniform cosmetic outcomes.

The consistently high satisfaction scores observed in the patient-reported outcomes, paired with early and clearly observable clinical benefits, suggest a synergistic effect between surgical technique and standardized postoperative wound management.

These results underscore the importance of integrating advanced technologies not only in the intraoperative phase but also in the postoperative continuum of care.

In this cohort of patients undergoing nipple-sparing mastectomy with immediate breast reconstruction, the application of the ***Novox^®^ Cup*** device was associated with favorable clinical outcomes and a positive impact in patient-reported wound-related quality of life. The device’s ease of use and tolerability contributed to a smooth postoperative recovery with minimal discomfort. Further studies are warranted to confirm these findings and to explore the potential of the ***Novox^®^ Cup*** in reducing local complications and improving the overall postoperative experience.

## 4. Discussion

In this retrospective observational study, the use of the ***Novox^®^ Cup*** device in the immediate postoperative period following nipple-sparing mastectomy (NSM) with immediate breast reconstruction proved to be safe, well-tolerated, and potentially effective in improving both clinical outcomes and patients perceived wound-related quality of life.

The ***Novox^®^ Cup*** is characterized by the combination of a gel-like oil matrix resulting from the oxygen-enrichment of natural oils, which is embedded in a polyester and polyurethane backing with a conformation that adapts in the most appropriate way to the treatment area (for example, as a breast cup or bra cup) [9].

The mechanism of action of the ***Novox^®^ Cup***, is based on a synergistic combination of ROS and carboxylic acid micro release and the oily nature of the matrix, which allow:

- the facilitation of the healing process by creating a microenvironment favorable to the activation of the microcirculation and consequently of the natural reparative processes of cell proliferation;
- the reduction in microbial contamination thanks to the ability to create a local microenvironment unfavorable to the proliferation of pathogens;
- the establishment of a protective barrier film with additional soothing action [9].

Skin flap and NAC viabilities remain a critical determinant of surgical outcomes in breast surgery, particularly in breast procedures where both oncologic safety and aesthetic preservation are paramount. Early identification of vascular compromise and timely intervention are essential to minimize complications and avoid secondary surgical procedures. The adoption of an internally standardized vitality algorithm allowed early identification of reversible vascular compromise (grade 2), which may represent the most clinically actionable subgroup in NSM surgery.

In the present cohort, the incidence of **total NAC necrosis (1.85%)** was low and consistent with rates reported in the breast surgery literature. Importantly, partial necrosis cases were successfully managed conservatively, highlighting the effectiveness of structured postoperative surveillance and advanced wound care strategies in preventing progression to more severe tissue loss. The overall local complication rate (30.4%) may appear elevated at first glance; however, the majority of recorded events were minor (Clavien–Dindo grade I–II) and did not require surgical intervention. When contextualized within published NSM benchmarks, where overall complication rates range between 20 and 40% depending on patient selection and reconstructive technique, our results fall within the expected spectrum. Importantly, major complications (Clavien–Dindo ≥ III) were rare, and only one patient required surgical reintervention.

It is important to note that the reported 30.4% complication rate includes minor events such as self-limited dehiscence or seroma not requiring operative management. When restricted to major complications (Clavien–Dindo ≥ III), the rate decreases to 1.8%, aligning with the high-volume NSM series.

No statistically significant association was observed between baseline variables and complication rates, although smokers showed a numerically higher incidence of grade ≥ 2 flap impairment.

A particularly relevant finding of this study is the outcome observed in **grade 2 flaps**, representing reduced but still acceptable vitality. These cases are clinically significant, as they constitute a potentially reversible condition that may either resolve or deteriorate depending on postoperative management. The **systematic application of Novox Cup dressings**, combined with closer clinical monitoring, resulted in **complete recovery in all grade 2 patients**, with no evolution toward grade 3 or 4 impairment.

This suggests that Novox Cup dressings may play a meaningful role in **supporting microcirculation, reducing edema, and promoting tissue recovery** in compromised yet viable flaps. Their use appears particularly beneficial in the early postoperative phase, when vascular instability is most pronounced and therapeutic intervention can alter the natural course of flap suffering.

Conversely, the small percentage of **grade 3 cases** underscores the importance of early recognition and aggressive management of moderate flap suffering. Prompt intervention in these patients is crucial to halt further ischemic damage and may explain the limited progression toward frank necrosis observed in this series. These findings are consistent with existing evidence regarding oxygen-enriched oil matrix-based dressings, which promote local microcirculation, reduce oxidative stress, and stimulate reparative cell proliferation [10].

The adoption of a **standardized vitality scale** proved useful not only for postoperative assessment but also for guiding treatment decisions and stratifying follow-up intensity. This approach enhances reproducibility, facilitates communication within the multidisciplinary breast team, and allows for more objective comparison of outcomes across studies.

In conclusion, our findings support the value of **early, standardized flap vitality assessment** in breast surgery and suggest that **targeted conservative interventions**, including the use of Novox Cup dressings, may significantly improve outcomes in patients with reduced but salvageable flap vitality.

The ***Novox^®^ Cup*** dressing plays a dual role in enhancing clinical wound healing and improving the patient’s recovery experience. When applied according to a standardized protocol—involving initial intraoperative placement, followed by patient-managed replacement at home—the device has been associated with smoother, faster, and more comfortable recoveries. Most patients resumed daily activities with minimal disruption, indicating benefits not only in physical rehabilitation but also in psychological well-being. Clinically, its use was linked to a reduction in complications such as flap necrosis or NAC ischemia—known challenges in breast-conserving mastectomy techniques.

Furthermore, the device’s capacity to establish a stable, antioxidant-rich microenvironment promotes more effective healing dynamics. This was reflected in both objective and subjective outcomes: patients consistently reported higher comfort levels, reduced scarring with more pliable and aesthetically favorable tissue, and better preservation of the NAC’s pigmentation and contour. These aspects are central to overall satisfaction, emphasizing the importance of integrating advanced technologies not only intraoperatively but throughout the postoperative care continuum [11].

Patient-reported outcomes, assessed using the validated ***Wound-QoL17 questionnaire***, confirmed minimal perceived burden in domains such as pain, exudate, sleep disruption, mobility, and emotional impact. The overall scores remained low throughout follow-up, suggesting that wound-related discomfort was limited. Descriptive analysis suggested an association between higher Wound-QoL17 scores and the presence of postoperative complications. Given the observational design and the primarily descriptive statistical approach, these findings should be interpreted as exploratory rather than confirmatory.

Importantly, only a small proportion of patients (1.6%) experienced delays of ≥2 weeks in initiating planned adjuvant therapies due to local wound complications. In our cohort, only one patient required a delay in adjuvant treatment, following the need to explant the prosthesis and replace it with a tissue expander due to a postoperative complication. This highlights the potential role of optimized postoperative management in maintaining oncologic timelines—a critical objective in the multidisciplinary treatment of breast cancer [12].

In summary, the combined clinical and patient-reported findings suggest that the ***Novox^®^ Cup*** dressing contributes positively to postoperative healing, reduces complications, and enhances patient comfort and satisfaction. These preliminary results warrant further validation through prospective controlled studies, but they provide a strong rationale for incorporating dressings into standardized postoperative protocols following NSM and immediate reconstruction.

Limitations include the retrospective design, absence of a comparator group, limited sample size, and use of a non-validated institutional vitality scale. Additionally, selection bias cannot be excluded, as patients with intraoperative evidence of compromised perfusion may have been more closely monitored.

The present findings should be interpreted as hypothesis-generating and supportive of further prospective controlled investigations.

## 5. Conclusions

Within the limits of an observational design, the integration of a structured vitality assessment protocol and an ROS-releasing dressing was associated with favorable short-term tissue outcomes. These findings justify further prospective controlled studies to determine whether such an approach may reduce progression from reversible flap suffering to necrosis in NSM surgery.

## Figures and Tables

**Table 1:** Skin flap vitality scale (1–4).

**1—Optimal vitality**
**Color:** Pink, Uniform
**Temperature:** warm and symmetrical with respect to the surrounding tissues
**Capillary refill:** <2 s
**Edema:** minimal or absent
**No signs of skin distress-->Perfused and stable flap**
**2—Reduced but acceptable vitality**
**Color:** slightly pale or hyperemic, but homogeneous
**Temperature:** slightly reduced or increased, but not critical
**Capillary refill:** 2–3 s
**Edema:** mild–moderate
**No necrosis**, any small areas of reversible distress--> Flap to be monitored more frequently, but vital
**3—Moderate Flap Suffering**
**Color:** marbled, cyanotic or grayish in patches
**Temperature:** clearly colder
**Capillary refill:** >3 s or absent
**Edema:** marked
**Areas of dubious vitality-->High risk of negative evolution, requires timely intervention**
**4—Necrosis or Severe Impairment**
**Color:** black, brown, dark purplish, or ischemic white
**Temperature:** cold
**Capillary refill:** absent
**Loss of sensation**, non-viable tissue
**Frank necrosis (partial or total)** = > Compromised flap: surgical evaluation required

**Table 2:** Clavien–Dindo classification.

**Grade I**
Complications that do not require pharmacological, surgical, endoscopic or radiological interventions.
Treatment with conservative therapies (e.g., analgesic, antipyretics, physiotherapy).
Not include antibiotics, medications, etc.
Example: Postoperative fever treated with antipyretics.
**Grade II**
Complications that require specific pharmacological interventions.
It includes antibiotics, blood transfusions, and parenteral nutrition.
Example: Infection treated with antibiotics.
**Grade III**
Complications requiring surgical, endoscopic or radiological intervention.
IIIa: Surgery performed without general anesthesia. Example: Drainage of an infected abscess/seroma/subcutaneous hematoma.
IIIb: Surgery performed under general anesthesia. Example: Re-surgery for hemorrhage.
**Grade IV**
Complications that endanger the patient’s life and require intensive management.

## Data Availability

The data supporting the findings of this study are available from the corresponding author upon reasonable request. Due to patient confidentiality and institutional policies, raw clinical data are not publicly accessible.

## Abbreviations

The following abbreviations are used in this manuscript:

NSM	nipple-sparing mastectomy
NAC	nipple–areola complex
ADM	acellular dermal matrices
ROS	reactive oxygen species
QOL	quality of life
